# I don't leave my people; They need me: Qualitative research of local health care professionals' working motivations in Syria

**DOI:** 10.1186/s13031-021-00432-y

**Published:** 2022-01-03

**Authors:** Agneta Kallström, Orwa Al-Abdulla, Jan Parkki, Mikko Häkkinen, Hannu Juusola, Jussi Kauhanen

**Affiliations:** 1 Institute of Public Health and Clinical Nutrition, Faculty of Health Sciences, The University of Eastern Finland, P.O. Box 1627, 70211 Kuopio, Finland; 2 Laurea University of Applied Sciences, Vantaa, Finland; 3 Department of Cultures, The University of Helsinki, Helsinki, Finland; 4 Independent Researcher, Helsinki, Finland

**Keywords:** Syria, Motivation, Health worker, Conflict, Violence, Qualitative, Experience

## Abstract

**Background:**

The Syrian conflict has endured for a decade, causing one of the most significant humanitarian crises since World War II. The conflict has inflicted massive damage to civil infrastructure, and not even the health care sector has been spared. On the contrary, health care has been targeted, and as a result, many health professionals have left the country. Despite the life-threatening condition, many health professionals continued to work inside Syria even in the middle of the acute crisis. This qualitative study aims to determine the factors that have motivated Syrian health professionals to work in a conflict-affected country.

**Methods:**

The research is based on 20 semi-structured interviews of Syrian health care workers. Interviews were conducted in 2016–2017 in Gaziantep, Turkey. A thematic inductive content analysis examined the motivational factors Syrian health care workers expressed for their work in the conflict area.

**Results:**

Motivating factors for health care workers were intrinsic and extrinsic. Intrinsic reasons included humanitarian principles and medical ethics. Also, different ideological reasons, patriotic, political and religious, were mentioned. Economic and professional reasons were named as extrinsic reasons for continuing work in the war-torn country.

**Conclusions:**

The study adds information on the effects of the Syrian crisis on health care—from healthcare workers' perspective. It provides a unique insight on motivations why health care workers are continuing their work in Syria. This research underlines that the health care system would collapse totally without local professionals and leave the population without adequate health care.

## Background

Health care workers (HCWs) everywhere face verbal and physical violence to a degree. In peaceful and stable societies their lives are rarely at risk. The perpetrators are commonly patients or private citizens [1]. Such aggression can have a negative impact on professionals' well-being and motivation. At worst it can put the provision of health care at risk or compromise its quality.

In fragile states and conflict settings, HCWs may also experience political and collective violence [2]. The warring parties may harm health care infrastructure, personnel, and logistics regardless of the International Humanitarian Law (IHL) that stipulates the healthcare system is protected in time of war [3, 4]. The United Nations Security Council (UNSC) has strongly condemned attacks against medical facilities and workers in UNSC resolution 2286 in 2016 [5].

Five years after the resolution was adopted, the number of HCWs killed in conflict settings in on the increase. In 2020 over 800 incidents of violence against health care were reported in conflict-affected countries and 185 HCWs were killed [6].

In Syria, the violence against health care has been alarmingly intense and used as a war strategy. The situation became worse after Russia joined the conflict in 2015 [7, 8]. From 2011 through March 2021, at least 930 HCWs have been killed. In most cases (55%), the primary cause of death is aerial bombardment or artillery fire by Government of Syria (GoS) and their Russian ally [8]. Health care facilities and ambulances have been subjected to airstrikes [9–11]. HCWs have been arrested, tortured, and executed by GoS. Other warring parties, such as numerous non-state armed groups (NSAGs)^1^ and the Islamic State in Syria and Iraq (ISIS), have also perpetrated against health care [8, 11–14].

Local Syrian HCWs working in highly stressful conditions are exposed to severe mental trauma, such as secondary traumatic stress and burn-out [15]. We have shown earlier that HCWs in Syria have specifically experienced violence due to their profession and may have lost relatives or even close family members [12].

Conflict situations cause qualified HCWs to depart their country [16, 17]. Mass migration of professionals and collapse of the health care system and infrastructure leave the civilians without adequate health care, thus increasing mortality and morbidity among the population [18–22]. The violence in Syria has been so intense that more than 70% of HCWS have migrated from the country. The figure is equal regardless of whether HCWs worked under ISIS rule in parts of Syria or NSAG controlled areas [12, 19, 22–24]. Our recent study points out that reasons for HCWs to leave to leave Syria were all related to security concerns [25]. The International Non-Governmental Organizations (INGOs) and most international organisations (IOs) have been forced to evacuate their non-local personnel due to security reasons leaving local workers to operate remotely across the neighbouring countries [26–28].

Due to the lack of qualified personnel, many HCWs who have stayed in Syria have had to work beyond their specialist academic training or experience. At the same time, 38% of physicians had less than 2 years of medical training. In addition, 38% of all HCWs had received no formal medical training at all. Significant variance exists between different regions. This lack of training and experience significantly increases the strain on the few remaining experts [23, 29]. Attempts at providing training on-spot use, e.g. through telemedicine for the HCWs, have been made [29, 30].

Despite the danger, some HCWs decide to risk their lives to provide health care services in a highly complicated and dangerous environment [23, 24]. This research aims to determine the motivations that influence the decision of HCWs to work in a country where the conflict has been raging for a decade.

Up to this point, only a small number of studies seek to understand why local HCWs decide to stay and work in active conflict settings with potentially life-threatening risks.

Although local HCWs usually bear the most significant responsibility and risk of the effects of war, the reviews on the motivations to operate in conflict settings have mainly focused on the motives of expatriates, especially those of Western humanitarian workers.^2^

The work motivation of humanitarian expatriates and locals may be different. The reasons for non-local workers vary from altruistic motives to adventure-seeking, while local humanitarian workers choose to help their communities and uphold humanitarian principles. However, there are often many different motives, and they usually overlap [31].

The research studying HCWs in conflict and post-conflict settings in northern Uganda identify several motivating factors such as community support, effective and flexible working conditions and, good leadership and communication [32].

In a study conducted in Yemen, a country with protracted violent conflict, fatalism and religious reasons were mentioned as essential motivations to work amidst war. In addition, Yemeni HCWs felt obliged to serve their countrymen and provide health care to them even when faced with peril [32].

Salary, job security, career advancement and attaining technical training are known motivators. Humanitarian agencies are considered reliable employers in damaged or deteriorating economic settings, offering relatively high payroll and employee benefits [31, 32].

We could not find previous research on the motivations of HCWs in Syria to establish a baseline for the study. The situation in Syria differs from many earlier conflicts because some of the HCWs operate from Southern Turkey, where they live with their families. They have obtained permission to enter Syria and perform humanitarian work there [12]. This delivery of humanitarian aid cross-border to Syria was authorised by UNSC in resolution 2165 and the mandate since then regularly renewed. The most recent renewal was done in July 2021. In southern Turkey lies the province of Gaziantep, one of the regional hubs for humanitarian organisations that deliver aid to Syria [33]. In the early years of the conflict, the role of HCWs in Syria was to maintain services and coordinate aid efforts. Their efforts allowed building the system of cross-border humanitarian activities when external contributors arrange the resources from abroad, and the remaining physicians facilitate their distribution inside the country [34].

## Methods

This research aims to determine the factors motivating Syrian HCWs to work and even risk their lives in a war-affected country. The main question was: "What are the motivations for the local HCWs to work in Syria?".

### Study setting

In total, 20 semi-structured interviews were conducted in Gaziantep, Turkey, between 2016 and 2017. Gaziantep was chosen because of its vicinity to the Northern Syrian border. Interviews in Syria were not possible due to security reasons.

Starting from Tunis in 2010, demonstrations against the repercussions of authoritarian rule spread across the Middle East. In Syria, civil protests against the regime were met with violence when government forces opened fire in Daraa, the southern parts of Syria, in March 2011.

The violence spread throughout Syria, and in July of 2011, an armed group, the Syrian Free Army (FSA), was established. Groups with radical Islamic backgrounds, such as the al-Qaeda linked Jabhat al-Nusra, were formed and gained influence mainly in the Northern part of the country [35]. In 2013, the Islamic State (IS, Daesh) became a significant force in Syria.

The Global Coalition against Daesh, led by the United States, started a military campaign in 2014 in Iraq and Syria to crush the organisation [36]. In September of the following year 2015, the Government of Russia began military intervention after an official request from the GoS [37]. IBy early 2014, three Kurdish majority regions in the north had declared an autonomous area and began instituting a new administration called ROJAVA [38]. In 2016 Kurdish Democratic Union Party and groups allied with it declared a federal area in the northeast, arguing that federalism is a future for Syria [39].

During the interviews in 2016 and 2017, the conflict in Syria was in a full armed-conflict stage. Military actions by GoS and Russia were common and severe human rights abuse frequent. At that time, Syria was divided between the four main warring factions (1) The Government of Syria and its allies, (2) Non-state armed groups with different ideological and political motivations, (3) Islamic State in Syria, (4) Kurdish Forces.

After 10 years of war, the GoS controls most of the country. However, regions in the Northern parts are governed by different factions. The GoS with Russian and Iranian allies and proxies, Turkey, Israel, and the United States operate or maintain military forces in the country. In addition, different NSAGs groups and proxies and IS have a presence in Syria [40]. One could argue that Syria is entering a post-conflict phase, even though the situation is very fragile, volatile, and varies between different areas. Those Syrian refugees returning to the country may face extrajudicial killings, torture, kidnappings, and sexual violence even if they have had security clearances from the government before returning [41].

Notably, violence against dissident crowds or groups in early 2011 is not a new phenomenon in Syrian domestic politics. For example, in 1982, the military troops besieged the city of Hama, where officials of the outlawed Sunni Islamist Syrian Muslim Brotherhood organisation lived. When the siege ended, an estimated 20,000 people were killed [42].

The Arab-nationalist Baath Party has led Syria since the 1963 coup. Especially under the presidency of Hafiz al-Assad and his son, Bashar, Syria has been relatively stable. Despite the country's relatively politically peaceful stability, the regime had strict control over its citizens. The state of emergency rule was declared after the coup.^3^ This legislation gave the security apparatus the power to justify arbitrary arrests and detentions and ban all oppositions. Even before the conflict, imprisonment, e.g. political reasons, torture and ill-treatment leading to death, was reported. Human rights defenders, as well as, anti-governmental groups were harassed and persecuted. The state monitored and controlled all forms of media, and the freedom of expression was strictly restricted. For dissident citizens, punitive laws were used [43].

The regime has tried to maintain that it has represented the whole Syrian national community, even though Alawis^4^ have always been overrepresented in the leadership positions in the state and the security apparatus, This fact has created a kind of dissonance between sectarian reality versus official ideology [38]. During the long Baath era, the nature of the regime has, in fact, changed significantly, even though its official character as secular and nationalist has remained intact. During the Assads' rule, Syria has developed into what has been described as a "presidential monarchy." Personality cult has been underlined, whereas the original socialist and secular character of the party-state has waned. In parallel, the introduction of neoliberal economic reforms has significantly shifted the social basis of the regime support from rural peasants to the (upper) middle class of the cities. Historically, the opposition to the regime has come mainly from Syrian Islamists, who even mounted an insurrection in the late 1970s and early 1980s. To counter radical Islamists, especially during Bashar's period, accommodation with moderate Islam increased while, at the same time, control of Islam continued. In practice, this policy undermined the traditional secularism of Syria [44]. What is a significant shift, official presidential rhetoric has become explicitly religious and anti-secular since 2011. It has been argued that Syrians have developed a new version of popular Syrian nationalism to counter the official state nationalism represented by the Baath party, especially during the uprisings. This new nationalism can be understood as a continuation of the national movement whose project was abolished by the Baathist coup in the early 1960s. Later on in the conflict, this new anti-dictatorial and practical version of nationalism have been increasingly challenged by the growing importance of religious, sectarian and tribal identities, in addition to the official state nationalism [45].

### Study design and data collection

A qualitative study design was used and based on semi-structured interviews (*n* = *20*) among Syrian HCWs who worked in the country during the conflict. Interviews were done by a female interviewer (AK) and two males (MH and OA).

We used a snowball sampling method (SSM) to access potential participants that may be in hiding because of the political sensitivity of the research and possible security threat against participants. SSM, also known as a chain referral, enables researchers to locate and contact hard-to-reach groups and individuals. SSM may help researchers to overcome the unwillingness to participate due to distrust [46].

We started two different separate SSM chains in Gaziantep, Turkey, to avoid selection bias. The selection criteria required participants to be over 18 years old, Syrian and qualified HCWs. We used the International Labor Organization's International Standards Classification of The Occupations (ISCO-08) for the criteria of HCWs [47].

We obtained permission to record the interviews from the participants. Hand-written notes were made during the session. Part of the interviews was conducted in English, part in Arabic. These interviews were later translated into English by a professional translator. The quality and correctness of the translation were confirmed by an independent translator using random extracts from the material. Interviews lasted between 30 and 90 min. When no new information emerged from the interviews, i.e., the data was saturated, we stopped collecting interviews.

### Measurement and analysis

The interviews were transcribed verbatim and triangulated with hand-written notes. All identifiable information that participants mentioned were deleted from the material to respect participants anonymity. First, we (AK, JP, OA) read through the transcripts to familiarise ourselves with the research data. After that, we divided motivations into intrinsic and extrinsic categories. Motivators can be divided into intrinsic or extrinsic. Intrinsic motivators arise from within the individual itself. They include motives and values such as empathy, altruism, and pride. These motivators are unaffected by external rewards and embraced only when a person finds them inherently valuable. Extrinsic motivators are generated from external rewards and include money and opportunities for employment, non-monetary material rewards, and non-material rewards, such as heightened social status and increased knowledge [48, 49].

Then the text was analysed using thematic content analysis and inductive reasoning. The transcripts were coded based on a topic, and the identified motivations were categorised into intrinsic and extrinsic and further divided into sub-groups.

Quotations were chosen to enrich the text and give a voice to the participants and illuminate the participants' motivations. They also validate the assertions of this study.

### Ethics considerations and informed consent

The participants were informed of the purpose and aims of the research. They had an opportunity to ask questions from the researchers. The participants gave verbal informed consent, and they were notified that they might refuse questions or withdraw from the interview at any time. No such data was requested that would allow the identification of the participants. Participants chose the location of the discussions.

One of the interviewers (MH), a psychotherapist, monitored the participants' well-being during the interviews. Participants were given contact details of the interviewers and were encouraged to contact the research team if they felt the any need for discussion. Anonymity and confidentiality were guaranteed to the participants.

The University of Eastern Finland Committee on Research Ethics approved an ethical permit for the study. The principles in the Declaration of Helsinki were observed.

## Results

In this research, we studied motivating factors which Syrian HCWs had while working in conflict settings in Syria.

The main categories of intrinsic and extrinsic motivations used in this study emerged from data analysis. Each of these categories was further split into two sub-categories. Intrinsic motivations include (1) humanitarian principles and medical ethics, and (2) ideological reasons (patriotic, political and religious). Extrinsic motivations consisted of (1) professional reasons and (2) financial issues. These results will be presented below with quotations.

### Sample characteristics

Of the 20 participants of this study, 18 were males, and 2 were females. The age range was 23–47 years, and the mean age was 37.5 years. The majority of the participants were born in Northern Syria, specifically in the Aleppo governorate (*n* = *12*). Additionally, several participants from different parts of Syria was reached. One of the participants was born abroad.

Most of the participants (*n* = *16*) were married at the time of the interview and had (n = 14) at least one child.

We interviewed mostly physicians (*n* = *13*) with a speciality (*n* = *9*). Other physicians (*n* = *4*) were generalists who had started their specialisation but had had to suspend their studies because of the war. Occupations of the interviewees included pharmacists (*n* = *3*), health service managers (*n* = *2*), a nurse, and a dentist.

The UN border crossing mandate allows humanitarian workers, including HCWs, across Turkey to Syria [33]. Most of the participants had migrated from Syria with their families and resided in Gaziantep. Only a few still lived in Syria. They went to Syria monthly and worked there from a few days up to 1 month. Some worked no longer as clinical physicians but as health service managers for different international non-governmental organisations (INGO) or local non-governmental organisations (NGOs).

A fraction of the interviewees were still living in Syria, and they all worked according to their educational background. Those NGOs and INGOs in which participants mainly worked in areas controlled by the Kurdish forces or non-state armed groups (NSAGs) in the Aleppo governorate. All participants had worked under GoS at the time of the beginning of the conflict, and later many participants had worked in IS-controlled areas before leaving the area (Table 1).

**Table 1:** Participants demographic

*Total participants*	*n* = *20*
*Gender*	
Male	18
Female	2
*Age range*	from 23 to 47 years
*Mean age*	37.5 years
*Place of birth (governorate)*	
Aleppo	12
Deir Ez-Zour	2
Raqqa	2
Hama	1
Homs	1
Idlib	1
Abroad	1
*Family status*	
Married	16
Children	14
*Profession*	
Physician with speciality	9
General practitioner	4
Pharmacist	3
Nurse	1
Dentist	1
Health care manager	2

### Intrinsic motivations to work in Syria

Participants indicated several coincident reasons for their choice to work in Syria. We analysed two intrinsic groups of motivations. Practically all participants named at least one reason from the four humanitarian principles (humanity, neutrality, impartiality, and independence) as their motivation for resuming their practice in Syria. The second group was ideological motivators, subdivided into a patriotic and political ideology and religious reasons.

### Humanitarian principles and medical ethics

The most significant reason for HCWs to work in the middle of the conflict was to fulfil the humanitarian principles and medical ethics. Improving people's lives and reducing suffering were among the most significant factors affecting HCW behaviour. Participants felt their professions granted them the ability to help others. They believed that it was their duty to alleviate the suffering of civilians and expressed a feeling of responsibility for them.

The main reason is the humanitarian aspect of my work. They [civilians] are suffering because of the conflict. We have the skills to cope with the conflict because we are educated. Elderly women, elderly men and children, those who cannot cope with this crisis in Syria. They need our help. (general practitioner)

I'm very sorry for patients that have lost their arms, their eyes, their legs… We have a lot of patients like this. There is a lot of disability. There is a lot of people living in camps in bad situations, in the summer, under the sunlight… in the winter, under the rain and snow. (speciality doctor)

It appears that many of the interviewees had genuine empathy for Syrian children. Most of the participants were parents themselves and were concerned about the possible effects of the conflict on the children's future.

Whenever I go to Syria, my biggest daughter sits with me and asks: Why do you want to go? Why you? Why not someone else? I think that it's the picture of children. You can never take it away from your memory – a small child in Syria. Whenever you enter [Syria], you will see the orphans, the children in the poor area with nothing. No education, no safe water, nothing. You ask: "Why is it like this?". When you go to help, it helps them, and it affects them. At least feeling that there is someone who wants to care about them. This motion keeps you going. (health service manager)

Many of the participants were threatened and sometimes targeted by different warring parties. They emphasised the need to protect the impartiality and neutrality of care provision. They noted that they would serve all those in need, despite the patients' political opinions or cultural background.

We can understand the motivation of the regime, we can understand the motivation of the opposition, but we don’t want to be between them… People are dying, but let the war continue. At least to stop those [hospital bombings]. To have a chance to work normally like any doctor in this world. (speciality doctor)

If I see a person needing medical help, I will give. I will give the medicine. I don't care about nationality, religion… (speciality doctor)

Some of the participants expressed practical motivators, such as maintaining the workings of the healthcare system, which would undoubtedly collapse without their contribution. They wanted to avoid leaving the population without any health services.

To continue to help, to treat the pediatric population. It's important to me that the IDPs [internally displaced person] there in the area that they receive some care. There's not enough care. It's medically underserved. There is a great population that needs to be helped. There is a shortage of pediatric specialists. There is a shortage of medical staff. I can continue to help. (nurse)

Some participants mentioned that they and their colleagues had been offered visas and grants to study or work abroad. However, the interviewees had refused to leave because they felt obliged to carry the burden left by those HCWs who chose to vacate the country.

Somebody should do it. Many doctors went to Germany. There are more doctors in Frankfurt than we have doctors in Aleppo. Syrian doctors. Who will do that [work]? (speciality doctor)

I want to give something to others. This is the meaning of being a doctor. Otherwise, there is no meaning. It's not only about money. Sometimes you have to have a meaning in your life. To touch this kind of humanitarian issue that we are facing here. This is the only thing that I can think about. (speciality doctor)

### Ideological reasons: patriotic, political and religious

Ideological reasons were divided into patriotic, political and religious causes to continue working. Many participants mentioned their love for their home country and the people as a significant reason to endure the hardships. They felt that they had to help civilians inside the country. Some even underlined their role as patriotic professionals.

They are my people. It is my country. It is my city. Somebody should do that. I know that it will be a risk. (general practitioner)

The reason that made me work in health care and continue my work; I was able to serve my people by giving health care in the middle of the war. (pharmacist)

Some participants mentioned political reasons; they said that they wanted democracy and human rights for all Syrians. Some participants described their role widely as pro-democracy and human rights activists even before the conflict started. Some had been under the surveillance of GoS already because of their political thoughts that the regime considered dangerous.

It was the year 2005—the day of the Declaration of International Human Rights. I participated in this [demonstration] with about 30 other students at the university. We just stand up. The day after, the security called my family, and I got a call from the military investigation to attend their office. (speciality doctor)

Many participants said that they actively participated in non-violent demonstrations and even organised them in the early stage of the conflict. These demonstrations were met with violence. Many protesters were beaten, detained or also killed by GoS. Still, some of the participants continued to be motivated by political ideology. They believed they had the responsibility to carry on the liberation of their country from Assad's regime.

The main reason that as an activist, I wanted to take part in the demonstrations. I see myself as responsible for my country. I see myself as responsible for the revolution to succeed. A lot of people have now been arrested; a lot of females are now in al-Assad's prisons. A lot of people have been killed. Me and all the other people in [name of the city retracted] in Syria are still trying to win this war, revolution to succeed. See yourself as responsible for all of that. We have to take responsibility for everything we have started with. That's why I am still there [in Syria]. (general practitioner)

Religious motivations were mentioned only by one of the participants.. Being a part of ummat al-Islām, i.e. the collective Islamic community, is a reason to continue work in challenging conditions in Syria.

We are brothers of Islam. There is a religious idea that we are serving our brother in religion, and we cannot turn our back from them. (speciality doctor)

### Extrinsic motivations

Additionally, participants expressed extrinsic motivation for their work in Syria. The participants mentioned economic and professional reasons.

### Economic reasons

In the initial stages of the conflict, few physicians dared to abandon their work at governmental hospitals in fear of losing their salaries. Money was one of the causes they remained in GoS controlled territory, even though some of them worked voluntarily and secretly at underground hospitals and networks providing health care services for those injured by GoS. They were careful to hide these activities. Those caught would be detained by GoS. In some cases, not even their family members were aware of their secondary jobs.

There wasn't any kind of financial support for doctors. We were all volunteering. If you want to have a stable income for your family, you have to stay in a hospital that really can give you a salary to survive. Some doctors stayed in hospitals. The decision was so hard to make. We had to work under the table, we had to have our channels, and then we started to practice, like helping a small number of people. [at the same time] we stayed in our hospitals. (general practitioner)

Several interviewees regarded economic reasons as important motivators behind their return to work in Syria. When the violence intensified, and the conflict escalated, many HCWs migrated outside Syria. Most of the participants settled with their families in Gaziantep, Southern Turkey. However, daily expenses were high, and the participants were unable to find any work. Working as a physician in Turkey was not an option. Turkish laws would require them to have a local license, which was not easily granted. Lack of funds forced them to consider working opportunities in Syria.

There's no work permission. I hire a small apartment here [in Turkey], it costs about three hundred dollars a month. This is without electricity and a mobile phone. It's a little bit expensive for the refugees here. (speciality doctor)

I need work because I need to survive with my family, to have income. I'm lucky because I have work [in Syria], and I can help my country at the same time. (speciality doctor)

Some participants mentioned the need to maintain their medical skills and knowledge for continuing their work. This was most commonplace among the doctors. Without the opportunity to practice their profession in Turkey, they feared their skills would diminish over time.

I'm a doctor. I should work. If you don't work for two years, you'll lose your ability to work, your ability to do surgeries. You lose your medical knowledge. You should stay in contact with health care [profession]. (speciality doctor)

## Discussion

This qualitative research studied the motivations for Syrian HCWs to work in the country. Our study adds to the small body of existing research on why local HCWs keep working during ongoing violent conflicts. It provides specific information on motivational factors that help Syrian HCWs to continue or even return to work amidst the war. This research creates a baseline study on the motivations of health care workers in Syria, since no previous studies have been conducted in the country, and overall the studies of this subject regarding contemporary conflicts are limited.

From the Syrian HCWs experiences, we identified both intrinsic and extrinsic motivators. Intrinsic motivations included humanitarian and medical ethics. These were the most common motivations among the participants, who felt they had a moral obligation, duty, and skills to help people in Syria. HCWs' feeling that they are the last chance for the civilians in the middle of the conflict, is also noted in the recent study conducted in Yemen [50].

Many participants emphasised the humanitarian values, such as impartiality and neutrality of their work. They HCWs wanted to stay out of the hostilities and only focus on saving lives. This is in accordance with Slim [31], who argues that one of the motivations for local workers is to uphold humanitarian principles. In addition to saving people's lives, HCWs felt that they had a moral obligation to prevent health care from collapsing entirely. Without their effort the civilians would likely have been left completely without health care in many parts of Syria.

Another intrinsic group of motivators was ideological reasons, which we divided into three different sub-categories (1) patriotical, (2) political, and (3) religious motivations. The patriotic motivations were most common, while religious motivations were, quite unexpectedly, less frequent and only mentioned by one participant.

In our research, patriotic reasons were a common motivator for the participants. Syrian nationalism has long roots in the modern history. However, the ideology among participants is not connected with the ruling Baath party nor president Bashar al-Assad, but the Syrian people themselves.

Slim [31] also argues that local humanitarian workers do their work to help their communities. As the case has been in Yemen [50], supporting local people is also a valid motivator among Syrian HCWs. The al-Assad rule's central doctrine has been unifying Syrian nationalism and loyalty to the Baath party indistinguishably. As many of the participants came under state persecution, they abandoned what little loyalty they might have had for the regime. Still, they continued to honour a tradition of patriotic pride separate from authoritarian notions. Many described their love for Syria as their home nation as a significant reason to continue helping the civilians during wartime. Those with political motivations had hopes of bringing about democracy and new nationalism to the country.

The political motivations are related to assisting the popular uprising. This approach is not primarily tied to their profession but rather an expression of their world views. Among other Syrians, medical professionals and students participated in and organised anti-government rallies [12].

Most interviews were conducted during June and July of 2016. At that time, GoS had not yet recaptured Eastern Aleppo. However, in autumn 2021, GoS controlled around 65–70% of the country. Approximately one-fourth of the country is governed by the Autonomous Administration of North and East Syria. In northwestern parts of the country, the Salvation Government, led by Hay'at Tahrir al-Sham and Turkish-backed factions, control around 5–10% of the territory [51]. After the interviews were done, GoS has successfully recaptured many major cities and strategically essential territories, rendering the uprising practically unviable. It's unlikely that today the hope for a successful popular revolution against the regime is a significant motivator for Syrian HCWs.

Economic reasons came up as one of the motivating factors. This result is consistent with the study conducted in conflict and post-conflict settings in Uganda [32]. The Syrian HCWs were not allowed to work in Turkey. The financial situation among refugees in Turkey was difficult in 2017. Nearly 64% of the Syrians lived below the poverty line [52]. NGOs and INGOs represent a reliable source of income [31], and the participants felt that working for such organisations was an opportunity for them to take care of their families. In 2021 the Syrian economy has deteriorated due to over a decade of war, economic malpractice and corruption, economic sanctions, and Lebanon’s deep financial crisis that has accelerated Syria’s economic collapse. The families in Syria cannot secure basic food rations or household items [53]. Today, financial motivations may have an even more substantial impact than at the time of interviews, especially among participants who live in Syria and gain their salary from NGOs or IOs.

Professional reasons were mentioned as motivating factors, especially among physicians. Without the opportunity to practice medicine, they were afraid to lose their skills. On the other hand, inexperienced physicians can gain valuable work experience in that situation. Professional training can be enhanced, for example, by distance learning, as has already been done to in Syria [29].

Religious motivations were mentioned only by one participant. This notable when compering to study conducted in Yemen where the religious motivations and fatalism were most common among Yemeni professionals [50]. The relative lack of open religious motivations may testify to the long-established secularism in modern Syria, which has been one of the central tenets of the official Ba’thist ideology. During the long tenure of Hafiz al-Assad (1970–2000), the earlier aggressive version of secularism was modified and the regime ended its earlier secularization agenda and religious discourse gained more presence in the public sphere. This trend has event strengthened during Bashar al-Assad’s Presidency. Yet, the acceptance of a basically secular order of the society and the concept of religion as a personal choice has remained [51].

## Strengths and limitations

This study has several notable contributions. This research is the first study trying to understand local HCWs working motivations during the conflict in Syria. The interviewing team consisted of people with different academic backgrounds. The interviewers were a female (AK) and two males (MH and OA). The non-Syrian interviewers (AK, MH) are familiar with working and interviewing persons in the Middle East and conflict settings. The researcher of Syrian origin (OA) is an experienced front-line health service provider. The results from all interviewers were almost identical, indicating that the methodology was efficient and the personality or background of the individual interviewers did not introduce systematic error. We also managed to reach Syrian HCWs for this study and gain their trust, and even the topic is politically susceptible and controversial.

These findings are subjected to limitations. The sample is not fully representative, and the results cannot be fully generalised to all Syrian HCWs. Due to the nature of snowball sampling methods, we did not have any HCWs working in the regime nor ISIS-controlled health facilities at the time of interviews. Due to the interviews in Southern Turkey, we reached primarily professionals working in the opposition-controlled Aleppo governorate. We also had a limited number of perspectives of women and their motives to work in Syria. It is also possible that healthcare workers currently operating in Syria are primarily men.

Having access only to HCWs on the opposition side is arguably the most distorting shortcoming. It's highly likely that professionals willingly operating under GoS would paint a more favourable picture of the regime. These HCWs may view loyalty to al-Assad as true patriotism and consider the opposition as traitors to Syria. Their motivations for working would most likely present an entirely different spectrum of reasons. Unfortunately, due to practical circumstances, they could not be interviewed for this study.

Similarly, HCWs supporting Islamistic NSAGs were not reached for this study, or participants did not express their affiliation to organisations. Such a group has most likely differing ideological and practical reasons to operate in war-zone and might have expressed treating wounded soldiers for strategic reasons as a significant motivation.

The woman perspective in the circumstances, where the difference between genders carries a high cultural significance, would undoubtedly be categorically different. In addition, having a higher number of religious participants could have revealed more about ideological motivations. It is also possible that participants did not want to discuss their religious reasons, and we did not ask about participants' beliefs. It remains unknown whether having more HCWs besides physicians or more participants from other territories would have changed some of the weights, such as the significance of financial or skill-maintenance relater motivators.

## Conclusions

Since the Syrian conflict has shown signs of de-escalation, restoring the civil infrastructure, including hospitals with qualified HCWs, has become topical. The war-induced challenges to health care in Syria are enormous. Reinstating health care functionality will be impossible without a sufficient number of qualified personnel. The COVID-19 pandemic and ongoing economic crisis has placed additional strain on the health care system. The political situation is very fragile overall, although some parts of the country can be considered to be in a post-conflict stage.

Many professionals have migrated from the country, but as this study indicates, HCWs have strong motivations to continue their work despite harsh conditions. HCWs want to fulfil humanitarian values and medical ethics. Their connection to the locals is strong. Despite these factors, it may be challenging to attract HCWs to work areas under GoS. The harsh rule of the regime may deter potential returnees. Some people who have voluntarily returned to the country have been persecuted. It likely that HCWs will not return to GoS controlled areas, if they fear for their life. In particular, those physicans who have ended up in the Western countries have likely integrated the new place. If they are able to practice their profession within the host country health care systems and their children have grown into these societies, they have little reason to return. Mere money or other extrinsic factors are are likely to be insufficient incentives for return, and do not bring solution insufficient to resolve the problem of understaffed health care.

To overcome such resistance and attarct the HCWs back to areas under GoS, understanding of their inner values and motivations is imperative. If returnees could be given credible guarantees for their safety, their willingness to consider moving back to Syria would likely increase.

The international community's responsibility is to ensure that international humanitarian law is followed and universal human rights are respected. This is a critical precondition for maintaining the provision of high quality health care services during conflicts. In order to proceed efficiently, the crisis dynamics of relevant human resources need to be properly understood. Studying the motivations of HCWs will provide the international community and researchers with much needed insight into these behauviors. The findings presented in this article can be developed into practical-level tools for protecting and restoring health care resources in complex humanitarian settings.

## Data Availability

The datasets generated and/or analysed during the current study are not publicly available due to security reasons but are available from the corresponding author on reasonable request.

## Abbreviations

GoR: The Government of Russia
GoS: The Government of Syria
HCW: Health care worker
INGO: International non-governmental organisation
ISIS: The Islamic State in Syria
NGO: Non-governmental organization
NSAG: Non-state armed group
UNSC: United Nations Security Council

## Footnotes

1 in this study we refer to non-state groups that includes all armed groups with different motivations and ideologies from Free Syrian Army to jihadist Hay´et al.-Tahrir. This definition excludes Islamic State (IS) and Kurdish Forces.

2 see Oberholster, A.J., Clarke, R., Bendixen, M. and Dastoor, B. (2013), "Expatriate motivation in religious and humanitarian non‐profit‐organizations", Journal of Global Mobility, Vol. 1 No. 1, pp. 7–27. https://doi.org/10.1108/JGM-09-2012-0007, Bjerneld M, Lindmark G, McSpadden LA, Garrett MJ. Motivations, concerns, and expectations of Scandinavian health professionals volunteering for humanitarian assignments. Disaster Manag Response. 2006 Apr-Jun;4(2):49–58. https://doi.org/10.1016/j.dmr.2006.01.002. PMID: 16,580,984.

3 the emergency rule was abolished after the demonstrations started in 2011.

4 “heterodox” Shiite.
